# Survey on the working, training, and research conditions of resident physicians in internistic and rheumatological continuing education—BEWUSST

**DOI:** 10.1007/s00393-023-01433-3

**Published:** 2023-10-17

**Authors:** Fabian Proft, Diana Vossen, Xenofon Baraliakos, Michael N. Berliner, Martin Fleck, Gernot Keyßer, Andreas Krause, Hanns-Martin Lorenz, Bernhard Manger, Florian Schuch, Christof Specker, Jürgen Wollenhaupt, Anna Voormann, Matthias Raspe, Martin Krusche, Alexander Pfeil, Kommission Fort- und Weiterbildung der Deutschen Gesellschaft für Rheumatologie

**Affiliations:** 1 Department of Gastroenterology, Infectiology and Rheumatology (including Nutrition Medicine), Charité – Universitätsmedizin Berlin, Berlin, Germany; 2 Rheumazentrum Ruhrgebiet, Ruhr-Universität Bochum, Herne, Germany; 3 Rheumatology and Geriatrics, Helios Klinikum Berlin-Buch, Berlin, Germany; 4 Clinic and Polyclinic for Internal Medicine I, University Hospital Regensburg, Regenburg, Germany; 5 Clinic and Polyclinic for Rheumatology/Clinical Immunology, Asklepios Klinikum Bad Abbach, Bad Abbach, Germany; 6 Department of Internal Medicine, Clinic for Internal Medicine II, University Hospital Halle, Halle (Saale), Germany; 7 Clinic for Internal Medicine, Department of Rheumatology, Clinical Immunology and Osteology, Immanuel Hospital Berlin, Berlin, Germany; 8 Division of Rheumatology, Department of Medicine V, University Hospital Heidelberg, Heidelberg, Germany; 9 Medical Clinic 3, Rheumatology and Immunology, University Hospital Erlangen, Friedrich-Alexander University Erlangen-Nuremberg, Erlangen, Germany; 10 Internal Practice Group Rheumatology-Nephrology, Erlangen, Germany; 11 Clinic for Rheumatology and Clinical Immunology, Evangelisches Krankenhaus Kliniken Essen-Mitte, Essen, Germany; 12 Immunologikum Hamburg, Hamburg, Germany; 13 German Society for Rheumatology, Berlin, Germany; 14 Department of Infectious Diseases and Pulmonary Medicine, Charité – Universitätsmedizin Berlin, Corporate Member of Freie Universität Berlin, Humboldt-Universität zu Berlin, and Berlin Institute of Health, Berlin, Germany; 15 Section for Rheumatology and Inflammatory Systemic Diseases, University Hospital Hamburg-Eppendorf (UKE), Hamburg, Germany; 16 Department of Internal Medicine III, University Hospital Jena, Friedrich-Schiller-University Jena, Am Klinikum 1, 07747 Jena, Germany

**Keywords:** Speciality of internal medicine and rheumatology, Compatibility of career and family, Compatibility of work and research, Perspectives, Fields of activity, Fachgebiet Innere Medizin und Rheumatologie, Vereinbarkeit von Beruf und Familie, Vereinbarkeit von Arbeit und Forschung, Perspektive, Tätigkeitsfelder

## Abstract

**Background:**

Data on the training and continuing education situation of residents in the field of internal medicine and rheumatology are not available for Germany. For this reason, the Commission for Education and Training of the German Society of Rheumatology (DGRh) initiated the BEWUSST survey on the working, training and research conditions of residents in rheumatology.

**Methods:**

A total of 102 questions on the topics of working conditions in everyday professional life, continuing medical education and training, compatibility of career and family, compatibility of work and research, perspectives as a rheumatologist and practical activities were included in an online questionnaire.

**Results:**

A total of 102 participants took part in the survey. Of the respondents 48.1% were satisfied with their professional situation, 40.2% of the participants were supervised by a specialist mentor and 54.9% were working as scientists during their work as a physician. A compatibility of family and career was possible for 34.7%. After completion of the residency 52.9% of the respondents aspired to a combined clinical and outpatient activity.

**Conclusion:**

Half of the trainee rheumatologists are satisfied with their professional activities, although mentoring of the assistants in training should be further improved. With respect to the desired combined clinical and outpatient activity, the existing options should be expanded or new professional fields of activity should be established, so that the specialty remains attractive for the upcoming generations.

**Supplementary Information:**

The online version of this article (10.1007/s00393-023-01433-3) contains supplementary information for the survey on the working, training, and research conditions of resident physicians in internistic and rheumatological continuing education—BEWUSST.

## Abstract (de)

**Hintergrund:**

Daten zur Aus- und Weiterbildungssituation von Assistenzärzt:innen des Fachgebietes Innere Medizin und Rheumatologie liegen für das Bundesgebiet nicht vor. Aus diesem Grund initiierte die Kommission Fort- und Weiterbildung der Deutschen Gesellschaft für Rheumatologie (DGRh) eine Umfrage zu den Arbeits‑, Weiterbildungs- und Forschungsbedingungen von Assistenzärztinnen und -ärzten in der rheumatologischen Weiterbildung.

**Methodik:**

Es wurden 102 Fragen zu den Themenkomplexen Arbeitsbedingungen im Berufsalltag, ärztliche Fort- und Weiterbildung, Vereinbarkeit von Beruf und Familie, Vereinbarkeit von Arbeit und Forschung, Perspektive als Rheumatolog:in und praktische Tätigkeiten über einen Online-Fragebogen gestellt.

**Ergebnisse:**

Insgesamt haben sich 102 Teilnehmer:innen an der Umfrage beteiligt; 48,1 % der Befragten waren mit der beruflichen Situation zufrieden, 40,2 % der Teilnehmer:innen wurden durch eine/n fachärztliche/n Mentor:in betreut, und 54,9 % der Teilnehmer:innen sind wissenschaftlich tätig. Eine Vereinbarkeit von Familie und Beruf war für 34,7 % möglich. Nach Abschluss der Facharztausbildung strebten 52,9 % der Befragten eine kombinierte klinische und ambulante Tätigkeit an.

**Diskussion:**

Die Hälfte der angehenden Rheumatolog:innen ist mit der beruflichen Tätigkeit zufrieden, wobei das Mentoring der Weiterbildungsassistent:innen eine weitere Verbesserung erfahren sollte. Hinsichtlich der gewünschten kombinierten klinischen und ambulanten Tätigkeit sollten die vorhandenen Optionen ausgebaut bzw. neue berufliche Tätigkeitsfelder etabliert werden, damit das Fachgebiet für den Nachwuchs attraktiv bleibt.

In recent years, the field of rheumatology and clinical immunology has experienced enormous progress in research, clinical practice and treatment. Through a better understanding of the pathophysiological processes in the immune system, a large number of innovative new therapies have been developed. In addition, treatment outcomes can be improved, and permanent damage to patients avoided through early diagnosis and appropriate treatment initiation.

However, German rheumatology, like other medical fields, will face special challenges in the coming years. Due to the foreseeable rising demand for care and the growing shortage of specialists, there is a risk of growing shortages in rheumatological care. The 2016 Memorandum of the German Society of Rheumatology (Deutsche Gesellschaft für Rheumatologie; DGRh) already called for a significant increase in the number of positions for rheumatology specialists to meet the growing demand a few years ago [18].

Unfortunately, however, there has been little structural change since then, and the number of new specialist positions has stagnated [14]. A survey performed in Saxony-Anhalt, Saxony and Thuringia by Keyßer et al. showed that half of the rheumatologists will reach retirement age in the next 10 years [4]. In view of the increasing prevalence of the diseases and the higher life expectancy of patients [6], the need for rheumatologists in Germany will continue to grow [1].

In an analysis from 2021, it was shown that a total of 17.2% of the training positions in the speciality remained vacant, which mostly concerns the outpatient sector (43.1% compared with 11.4% of the clinical sector) with unavailable financing [12]. This gap needs to be closed to counteract this downward trend.

This requires, among other things, attractive training conditions that are oriented towards uniform standards [13] and offer opportunities for modern working time and career models. Initial small-scale work in recent years has already shown potential for improvement [5, 10].

In order to systematically analyse the current training and continuing medical education situation and to identify potential areas for improvement, the Commission for Continuing Education and Training of the DGRh therefore initiated the BEWUSST survey (*Be*fragung zu den Arbeits-, *W*eiterbildungs- und Forsch*u*ngsbedingungen von A*ss*istenzärztinnen und -ärzten in der internistisch-rheumatologischen Wei*t*erbildung; Survey on the working, training, and research conditions of resident physicians in internistic and rheumatological continuing education) on the working, training and research conditions of residents in continuing medical education for internal medicine and rheumatology in 2022. For better comparability with other disciplines, the surveys by Raspe et al. of the German Society of Internal Medicine (Deutsche Gesellschaft für Innere Medizin; DGIM) were used as a basis [15, 16]. Here, a survey of the continuing education situation in general internal medicine was conducted in 2016.

In addition, the BEWUSST survey also addresses other subject-specific aspects of the rheumatology training curriculum, such as immunological laboratory diagnostics and rheumatological examination techniques.

## Methods

The questionnaire was created using the QuestionPro system (QuestionPro GmbH, Berlin, Germany). All questions could be answered online between May 4, 2022 and October 31, 2022. The survey was advertised via the DGRh newsletter and the social networks of the Young Rheumatology Working Group (Arbeitsgemeinschaft Junge Rheumatologie; AGJR). All rheumatologists authorised to provide rheumatological training received a separate letter raising awareness for the survey and motivating them to take part.

The survey included a total of 102 questions on the following topics: basic data (*n* = 15), working conditions in everyday professional life (*n* = 5), continuing medical education and training (*n* = 17), compatibility of work and family (*n* = 10), compatibility of work and research (*n* = 16), perspectives as a rheumatologist (*n* = 14), practical activities (*n* = 22) and personal opinions/comments (*n* = 3).

After completion of the evaluation, the survey results were extracted from the QuestionPro system and transferred to an Excel spreadsheet (Microsoft Excel 2016, Redmond, WA, USA). All data were available as absolute numbers or percentages.

### Ethics

The data analysis was carried out according to the regulations of the Ethics Committee of the University Hospital of Jena, Germany.

### Statistics

After summarising the data, descriptive statistics were performed. The statistical analyses were carried out with the software IBM SPSS Statistics version 27.0 (IBM Corp., Armonk, NY, USA) for Windows.

## Results

### Basic data

A total of 102 trainees (women *n* = 68 [66.7%] and men *n* = 34 [33.3%]) took part in the survey. The main age ranged from 30 to 34 years (40.2%). The majority of participants worked in the German federal states of Bavaria (20.6%) and North Rhine-Westphalia (19.6%). In 38.2% of the respondents, 2 children were living in the household; 91.2% of the participants had not finished any specialization.

**Fig. 1:**
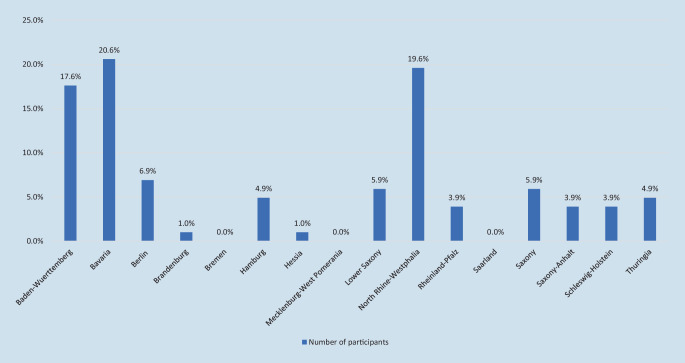
Percentage of participants per federal state

A total of 46.1% and 33.3% of the participants worked on a normal ward and in a hospital outpatient department, respectively. In 71.6% of the respondents, the hospital was in public ownership; 47.1% worked at a university clinic. The primary career goal of 38.1% of the participants was to establish a private practice, 27.3% aimed to become a senior physician at a hospital, 16.5% to pursue an academic career with habilitation/professorship and 3.4% to become a department head/chief physician (Fig. 1 and Table 1).

**Table 1:** Basic data of the survey participants

Participants	Total	*n* = 102
	Women	*n* = 68 (66.7%)
	Men	*n* = 34 (33.3%)
	Diverse	*n* = 0 (0%)
Age	20–24 years	*n* = 0 (0%)
	25–29 years	*n* = 23 (22.6%)
	30–34 years	*n* = 41 (40.2%)
	35–39 years	*n* = 31 (30.4%)
	40–44 years	*n* = 3 (2.9%)
	> 45 years	*n* = 4 (3.9%)

### Working conditions in everyday professional life

The survey revealed that 36.3% and 11.8% of the participants were “rather satisfied” or “very satisfied” with their professional situation. The factor most frequently causing dissatisfaction was a heavy workload (14.6%), without an exact definition of working time. The proportion of activities in the daily work routine was as follows: 42.7% work with and on patients, 31.8% patient-related work and 25.5% non-medical or non-patient activities (Table 2).

**Table 2:** Working conditions in everyday professional life

Question	Possible responses	Number (percent)
How satisfied are you overall with your current professional situation?	Very dissatisfied	*n* = 10 (9.8%)
	Rather dissatisfied	*n* = 17 (16.7%)
	Undecided—partly dissatisfied, partly satisfied	*n* = 26 (25.4%)
	Rather satisfied	*n* = 37 (36.3%)
	Very satisfied	*n* = 12 (11.8%)
What factors cause you dissatisfaction? (Up to three answers possible)	High workload	*n* = 41 (13.8%)
	Irregular working hours and shift work	*n* = 13 (4.4%)
	Work intensification	*n* = 26 (8.8%)
	Poor reconciliation of work and family life	*n* = 13 (4.4%)
	No time for research	*n* = 17 (5.7%)
	My private life suffers	*n* = 21 (7.1%)
	No independent medical decisions possible	*n* = 2 (0.7%)
	Strong economic influence on medical decisions	*n* = 16 (5.4%)
	Poor working atmosphere	*n* = 5 (1.7%)
	Rigid hierarchies	*n* = 4 (1.3%)
	High proportion of non-physician activities	*n* = 35 (11.8%)
	Poor quality of continuing medical education	*n* = 13 (4.4%)
	Lack of guidance and supervision	*n* = 19 (6.4%)
	Low recognition for work done	*n* = 13 (4.4%)
	Poor organisation of procedures/bad agreements	*n* = 26 (8.8%)
	Lack of prospects	*n* = 26 (8.8%)
	Other: free text	*n* = 7 (2.4%)

### Continuing medical education and training

A total of 75.5% of participants worked fulltime and 24.5% parttime. Of the parttime colleagues, 60% felt that they were disadvantaged in their continuing medical education and training. A total of 81.4% of the participants were employed at an institution with full authorisation for training as a specialist in internal medicine and rheumatology; 36.3% of the respondents had primarily received an employment contract for the entire period of their specialization; 22.5% stated that a structured training curriculum was available at their institution and that a transparent presentation of rotations was provided by the hospital management (42.2%). Training was completed within the scheduled time by 68.6% of participants. The use of external training opportunities was considered helpful by 55.9% of participants (Table 3).

**Table 3:** Continuing medical education and training

Question	Possible responses	Number (percent)
Do you currently work fulltime or parttime?	Fulltime	*n* = 77 (75.5%)
	Parttime	*n* = 25 (24.5%)
Do you have a structured training curriculum with learning content/rotations planned from the beginning?	No	*n* = 79 (77.5%)
	Yes	*n* = 23 (22.5%)
Will you be able to complete your training in the prescribed period of time (e.g., 5 years for internal medicine)?	No	*n* = 32 (31.4%)
	Yes	*n* = 70 (68.6%)

### Compatibility of career and family

The following results were obtained regarding satisfaction with the compatibility of career and family life: 6.9% “fully agree”, 27.8% “rather agree”, 29.2% “partly agree” and 22.2% “somewhat disagree”. Regarding the shift of tasks in favour of work (23.6% “fully agree”, 40.3% “rather agree” and 23.6% “partly agree”) and the support from colleagues (12.5% “fully agree”, 36.1% “rather agree” and 29.2% “partly agree”), a similar picture emerged. According to the participants, the flexible organisation of working hours (21.4%), home office (19.9%), less overtime (19.4%), better planning of working hours (14.4%), and a childcare place (4.5%) or all-day care (4.0%) led to an improvement in the balance between work and private life (Table 4).

**Table 4:** Compatibility of career and family

Question	Possible responses	Number (percent)
The following questions on the compatibility of work and family are primarily aimed at physicians with children. But even if you do not have a child/children, you are welcome to answer the following questions	I have a child/children	*n* = 37 (36.3%)
	I do not have a child/children, but would still like to answer the questions on work and family	*n* = 35 (34.3%)
	I do not have a child/children and would like to skip the questions on family and career	*n* = 30 (29.4%)
I am satisfied with the compatibility of family and work at my workplace	Fully agree	*n* = 5 (6.9%)
	Rather agree	*n* = 20 (27.8%)
	Partly agree	*n* = 21 (29.2%)
	Somewhat disagree	*n* = 16 (22.2%)
	Disagree	*n* = 10 (13.9%)
Consideration for employees with family obligations is often at the expense of employees without family obligations	Fully agree	*n* = 29 (40.3%)
	Rather agree	*n* = 20 (27.8%)
	Partly agree	*n* = 16 (22.2%)
	Somewhat disagree	*n* = 4 (5.6%)
	Disagree	*n* = 3 (4.2%)
Did you take parental leave?	I have not taken parental leave yet	*n* = 41 (56.9%)
	I have already taken parental leave	*n* = 31 (43.1%)
Which of the following factors would facilitate/enable you to achieve a good balance between work and a private life with family responsibilities? (Three most important factors)	More flexible working hours, e.g., by having more say in the determination of working hours	*n* = 43 (21.7%)
	More predictable or regular working hours	*n* = 29 (14.6%)
	Less overtime	*n* = 39 (19.7%)
	Childcare place	*n* = 9 (4.5%)
	All-day childcare	*n* = 8 (4.0%)
	Childcare place near the workplace	*n* = 4 (2.0%)
	More consideration from colleagues	*n* = 2 (1.0%)
	Carry out parts of the work at home (documentation, doctor’s letters via intranet/VPN)	*n* = 40 (20.2%)
	Optional childcare service for children or relatives in emergencies, for meetings or during the school holidays	*n* = 9 (4.5%)
	Financial support	*n* = 4 (2.0%)
	Mentoring programme	*n* = 5 (2.5%)
	I am satisfied with the current offer	*n* = 6 (3.0%)

*VPN* Virtual private network

### Compatibility of work and research

In total, 69.6% of the participants had a doctorate, of whom 54.9% continued to work in science. The scientific work mainly concerned clinical topics (73%). Scientific work was primarily carried out after regular working hours (71.6%). On average, the participants were listed as coauthors of one scientific paper per conference. Over a quarter (28.4%) of the respondents were aiming for habilitation. Furthermore, 61.8% of the participants were involved in student teaching (Table 5).

**Table 5:** Compatibility of clinical work and research

Question	Possible responses	Number (percent)
Are you currently working as a scientist or do you plan to do so in the future?	No	*n* = 46 (45.1%)
	Yes	*n* = 56 (54.9%)
What is the scientific field of your research work? (Multiple answers possible)	Experimental (basic research)	*n* = 21 (18.3%)
	Clinically orientated	*n* = 84 (73.0%)
	Epidemiological	*n* = 10 (8.7%)
When do you carry out your scientific work?	During working hours, in addition to the clinical activity	*n* = 27 (26.4%)
	In the free time following clinical activity	*n* = 73 (71.6%)
	There is a leave of absence to carry out the research work	*n* = 2 (2.0%)
Are you involved in student teaching?	No	*n* = 39 (38.2%)
	Yes	*n* = 63 (61.8%)

### Perspectives as a rheumatologist

For 77.5%, training in the outpatient sector was of interest. In the private practice sector, 13.7% of the respondents had completed a training period. After completing their specialist training, 52.9% wanted to combine clinical and outpatient work; 64% planned to work as employees. For 74.5%, working in a medical care centre as an employed physician was an option; 82.4% of the colleagues had not yet had any contact with a resident rheumatologist (Tables 6 and 7).

**Table 6:** Perspectives as a rheumatologist

Question	Possible responses	Number (percent)
Is specialist training in the outpatient sector of interest?	No	*n* = 23 (22.5%)
	Yes	*n* = 79 (77.5%)
Have you already completed training in the private practice sector?	No	*n* = 88 (86.3%)
	Yes	*n* = 14 (13.7%)
Would you like to work in a clinical or outpatient setting?	Clinic	*n* = 13 (12.7%)
	Outpatient setting	*n* = 35 (34.3%)
	Clinical and outpatient setting	*n* = 54 (52.9%)

**Table 7:** Professional life as a specialist in internal medicine and rheumatology

Question	Possible responses	Number (percent)
Work–life balance	1 (very important)	*n* = 38 (37.3%)
	2	*n* = 33 (32.4%)
	3	*n* = 9 (8.8%)
	4	*n* = 8 (7.8%)
	5	*n* = 5 (4.9%)
	6	*n* = 5 (4.9%)
	7	*n* = 4 (3.9%)
	8 (not important)	*n* = 0 (0%)
Parttime work	1 (very important)	*n* = 16 (15.7%)
	2	*n* = 12 (11.8%)
	3	*n* = 11 (10.8%)
	4	*n* = 12 (11.8%)
	5	*n* = 13 (12.7%)
	6	*n* = 12 (11.8%)
	7	*n* = 9 (8.8%)
	8 (not important)	*n* = 17 (16.7%)
Compatibility of family and work	1 (very important)	*n* = 44 (43.1%)
	2	*n* = 28 (27.5%)
	3	*n* = 9 (8.8%)
	4	*n* = 8 (7.8%)
	5	*n* = 5 (4.9%)
	6	*n* = 3 (2.9%)
	7	*n* = 5 (4.9%)
	8 (not important)	*n* = 0 (0%)
Clinical activity	1 (very important)	*n* = 26 (25.5%)
	2	*n* = 20 (19.6%)
	3	*n* = 22 (21.6%)
	4	*n* = 12 (11.8%)
	5	*n* = 5 (4.9%)
	6	*n* = 8 (7.8%)
	7	*n* = 8 (7.8%)
	8 (not important)	*n* = 1 (1.0%)
Self-employment	1 (very important)	*n* = 14 (13.7%)
	2	*n* = 20 (19.6%)
	3	*n* = 18 (17.6%)
	4	*n* = 13 (12.7%)
	5	*n* = 10 (9.8%)
	6	*n* = 11 (10.8%)
	7	*n* = 7 (6.9%)
	8 (not important)	*n* = 9 (8.8%)
Regional binding	1 (very important)	*n* = 14 (13.7%)
	2	*n* = 13 (12.7%)
	3	*n* = 16 (15.7%)
	4	*n* = 17 (16.7%)
	5	*n* = 12 (11.8%)
	6	*n* = 11 (10.8%)
	7	*n* = 10 (9.8%)
	8 (not important)	*n* = 9 (8.8%)
No weekend/night duty	1 (very important)	*n* = 39 (38.2%)
	2	*n* = 22 (21.6%)
	3	*n* = 11 (10.8%)
	4	*n* = 9 (8.8%)
	5	*n* = 5 (4.9%)
	6	*n* = 6 (5.9%)
	7	*n* = 1 (1.0%)
	8 (not important)	*n* = 9 (8.8%)
Earning potential	1 (very important)	*n* = 23 (22.5%)
	2	*n* = 26 (25.5%)
	3	*n* = 16 (15.7%)
	4	*n* = 16 (15.7%)
	5	*n* = 12 (11.8%)
	6	*n* = 1 (1.0%)
	7	*n* = 4 (3.9%)
	8 (not important)	*n* = 4 (3.9%)

### Practical activities

At the time of the survey, 91.2% of the participants had not yet completed their specialist training; 40.2% of the participants were supervised by a mentor (specialist in internal medicine and rheumatology) at their workplace. Arthrosonographies were performed under supervision or independently by 41.2% and 86.3% of the colleagues, respectively. The following data were collected for vascular ultrasound: under guidance 35.3% versus independently 43.1%. More participants, 60.8% and 73.5%, were able to perform joint punctures under guidance or independently, respectively. Regarding capillary microscopy, 29.4% of colleagues carried out the examination under supervision (54.9% independently). In total, 53.9% had the opportunity to acquire competence in rheumatological/immunological laboratory diagnostics at their own training centre, and 30.4% of the participants estimated that they already had decision-making skills in laboratory diagnostics at the time of the survey. Regarding radiological and nuclear medicine imaging procedures, the respondents had already received training in the following procedures: 48.0% X‑ray diagnostics, 35.3% computer tomography, 38.2% magnetic resonance imaging and 29.4% nuclear medicine imaging procedures (Table 8).

**Table 8:** Practical activities

Question	Possible responses	Number (percent)
Are practical activities in the workplace supervised by a mentor (specialist)?	No	*n* = 41 (40.2%)
	Yes	*n* = 61 (59.8%)
Do you perform arthrosonographies independently?	No	*n* = 14 (13.7%)
	Yes	*n* = 88 (86.3%)
Do you perform vascular sonographies independently?	No	*n* = 58 (56.9%)
	Yes	*n* = 44 (43.1%)
Do you perform joint punctures independently?	No	*n* = 27 (26.5%)
	Yes	*n* = 75 (73.5%)
Do you perform capillary microscopy independently?	No	*n* = 46 (45.1%)
	Yes	*n* = 56 (54.9%)

## Discussion

The BEWUSST survey focuses on the working, training and research conditions of resident physicians in continuing medical education in rheumatology. The results of the survey presented here provide important insights into the situation of colleagues in continuing medical education. A total of 102 participants from all over Germany took part in the survey. In the 2022 survey on training positions, 478 positions for internal medicine and rheumatology were evaluated, of which 82.8% (*n* = 396) were filled [14], so approximately 25% of residents for rheumatology in Germany took part in the survey.

It should be noted that half of the respondents worked at a university hospital, so the statements regarding training positions in the non-university sector are possibly insufficiently represented. On the other hand, university hospitals have the highest number of clinical training positions (45%; 177 of 391 clinical training positions) [12].

Two thirds of the participants were female, and over 90% were under 40 years of age. Three quarters worked fulltime, and one quarter parttime. Comparable results were found in the surveys of the German Society of Internal Medicine (fulltime 87% and parttime 13%), the German Radiological Society (fulltime 83% and parttime 17%), the Association of Residents in Training for Urology (fulltime 90% and parttime 10%) and the Society of Gynaecology and Obstetrics (fulltime 70% and parttime 30%) [2, 7, 9, 16].

Due to the anonymity of the survey, a representative picture of the current situation of residents in rheumatology training can be assumed. It should be emphasised as positive that almost half of the participants (48.1%) expressed satisfaction with their current work situation. This is to be interpreted in particular against the background of a higher level of satisfaction of the respondents compared to the results in internal medicine (38%), urology (44%) and gynaecology (40%) [2, 7, 16]. In addition to the high workload, the proportion of non-physician activities and the impact on personal life were cited as factors that negatively affect the work situation. The highest priority was given to the issue of reconciling family and private life with working life. Another important result of the survey is that residents in rheumatology training stated that they suffer from considerable work pressure and lack of time. As this can have a negative impact on their physical and mental health and lead to burnout and other health issues, it seems important to take measures to improve the work–life balance and to provide better support systems for residents.

It should be emphasised that more than three quarters of the participants stated that there is no structured training curriculum with plannable learning content/rotations at their training location. Again, the results of the current survey are in line with the previously published survey results for general internal medicine (78%), radiology (63%), urology (70%) and gynaecology (82%) [2, 7, 9, 16]. This seems to be a concern with a need and the potential for optimisation. The model curriculum of the DGRh can serve as a basis for the implementation of standardised and structured training in the field of internal medicine and rheumatology in Germany [13]. The implementation of a training curriculum is associated with an increase in satisfaction [16]. The additional use of the courses of the rheumatology training academy can further improve rheumatologically education in clinics and practices.

Overall, 70% of the respondents were doctoral graduates—with a significantly higher proportion than in internal medicine (52%), radiology (59%), urology (44%) and gynaecology (54%) [2, 7, 9, 15]. Moreover, 55% of the respondents are active as scientists (internal medicine 19%, radiology 51%, urology 39% and gynaecology 42%) [2, 7, 9, 16] and work mainly in clinically oriented research areas. However, the high proportion of scientifically active training residents must be seen against the background of a relative overrepresentation of participants from university institutions in our survey. Primarily (72%), scientific work is done after working hours. To strengthen scientific work, research periods should be established, with time off from clinical obligations.

The survey of the AGJR in 2019 indicated that only 19% of respondents reported regular mentoring [5]. In the current survey, this was true for 40% of the participants, which means that the situation has improved compared to the AGJR survey. However, the conditions regarding mentoring for the performance of rheumatological diagnostics (e.g., joint punctures) should be further improved to enhance the quality of training.

After completion of specialist training, half of the respondents aspired to a combined in- and outpatient activity, which should primarily be carried out as salaried employees; 74% of the survey participants see their future in a position in a medical care centre. These data are comparable to the results of a survey of rheumatology training residents from 2020 in Saxony-Anhalt, Saxony and Thuringia [10]. Taking over an independent rheumatology practice does not seem to be a desirable career alternative for many [10].

According to the survey, compatibility of family and career, namely the work–life balance, possibly as a parttime employee, is an important factor for professional activity. In the implementation of a combined in- and outpatient activity and, e.g., parttime employment, working in a medical care centre is an option for professional activity. Furthermore, outpatient specialist care forms an additional supplement for rheumatology training, since combined in- and outpatient practice is possible. To summarise, appropriate professional offers should be created for future specialists so that rheumatology remains attractive for training residents. The choice of an internal medicine speciality is often made at the end of medical school, and the possible lifestyle in the corresponding speciality plays an important role in the choice of speciality in many cases [3, 8]. In this context, the lack of independent rheumatology chairs or rheumatology departments at many university hospitals in Germany is critical, as good teaching during students’ medical education will clearly motivate young colleagues to choose this speciality for continuing medical education [11, 17].

In addition, such a survey should be repeated at regular intervals to be able to evaluate the effect of changes in training and, if necessary, to carry out a renewed adaptation.

## Conclusion

In summary, important findings can be drawn from the results of the BEWUSST survey that can lead to adjustment and continuous optimisation of rheumatological training education in Germany, incorporating the perspective of young colleagues in training. Consideration of the abovementioned negative implications is needed in light of an increasing shortage of internal rheumatologists and the rising prevalence of inflammatory rheumatic diseases. In order to ensure continued adequate rheumatological care, the number of training positions must be increased urgently.

## Conclusion for clinical practice

- Half of the participating future rheumatologists are satisfied with the working conditions in the specialty.
- Mentoring should be further expanded, e.g., by using the model curriculum with the implementation of a structured training programme.
- With regard to the preference for combined clinical and outpatient work, the existing options (e.g., outpatient specialist care) should be expanded or new professional fields of activity established so that the specialty also remains attractive for the next generation.

### Supplementary Information

Raw data from the survey on the working, training and research conditions of resident physicians in internistic and rheumatological continuing education—BEWUSST

## Abbreviations

AGJR: Arbeitsgemeinschaft Junge Rheumatologie; Young Rheumatology Working Group
DGIM: Deutsche Gesellschaft für Innere Medizin; German Society of Internal Medicine
DGRh: Deutsche Gesellschaft für Rheumatologie; German Society of Rheumatology
VPN: Virtual private network

## Declarations

This retrospective data analysis was conducted according to the regulations of the Ethics Committee of the University Hospital of Jena, Germany.
